# Recurrent Lateral Ventricular Enterogenous Cyst: A Report of an Extreme Rare Case

**DOI:** 10.1155/2017/3098676

**Published:** 2017-05-18

**Authors:** Md. Shamsuzzaman Mondle, Md. Shamsul Alam, Misbah Uddin Ahmad, Md. Abdullah Yusuf

**Affiliations:** ^1^Department of Neurosurgery, Shaheed Taj Uddin Ahmed Medical College, Gazipur, Bangladesh; ^2^Department of Neurosurgery, Bangabandhu Sheikh Mujib Medical University, Dhaka, Bangladesh; ^3^Department of Neurosurgery, National Institute of Neurosciences & Hospital, Dhaka, Bangladesh; ^4^Department of Microbiology, National Institute of Neurosciences & Hospital, Dhaka, Bangladesh

## Abstract

The patient was a 45-year-old man with a progressive headache. Evaluation in detail revealed it as a case of left lateral ventricular space occupying lesion (SOL) resembling choroid plexus papilloma. A left parietal craniotomy was done and the lesion was removed completely through intraparietal approach. Surgical removal resulted in complete symptomatic relief. Histopathology revealed that it was a case of the enterogenous cyst. One year after surgery, the patient again experienced the same symptom and the images revealed recurrence of the lesion. The patient has undergone 2nd surgery and histopathology was the same as before. The patient was given radiotherapy and now he is completely relieved and well. Although intracranial enterogenous cyst is not uncommon, intraventricular enterogenous cyst as well as recurrent intraventricular enterogenous cyst is a rare entity.

## 1. Introduction

Enterogenous cysts of CNS are unusual cystic lesions which have an epithelial lining resembling gastrointestinal mucosa [1]. These most frequently occur in the spinal canal, when they are well recognized [2]; however, they are the uncommon cause of spinal cord compression and can occasionally pursue a fluctuating clinical course which may mimic multiple sclerosis [3]. Anatomically these occur most frequently near the junction of the cervical and thoracic spinal cord, particularly in young patients, and are often associated with developmental vertebral anomalies. Only two previous case reports in the literature describe intracranial enterogenous cysts, although other reports describe such lesions in the high cervical region [4] and around the foramen magnum [5]. No previous reports of patients with either multiple enterogenous cysts or with such cysts above the tentorium are reported. The developmental origin of such cysts has been discussed previously and a number of theories have been proposed about their etiology although this issue is still unresolved.

## 2. Case Presentation

The patient was a 45-year-old man who presented with a headache for 3 months which was progressive and had been intolerable for 2 weeks. However, the patient was a football player with good health. There was no such family history and family members were perfectly well. There were no other clinical symptoms reported by the patient.

**Figure 1:**
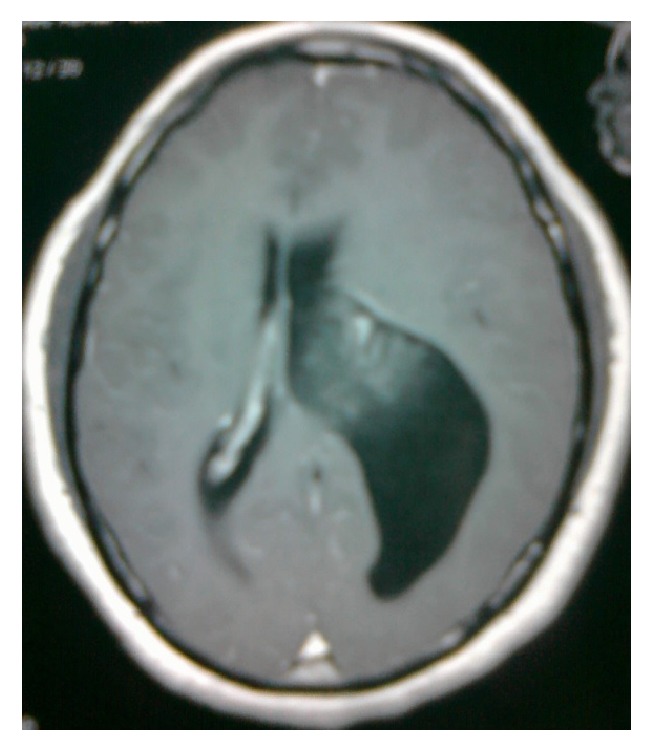
Brilliantly enhancing lesion in left lateral ventricle on MRI.

The patient was advised to do CT-scan and MRI. Interestingly both CT-scan and MRI revealed brilliantly enhancing left lateral ventricular lesion with unilateral hydrocephalus (Figure 1). T1 Image axial view postcontrast (Figure 3) and precontrast film (Figure 4) were also examined. T1 image of coronal view in postcontrast film was also evaluated (Figure 5). T2 image in sagittal view (Figure 6) also found the SOL.

Flare image (Figure 7) was examined as well. All other investigations were within normal limit. Then the patient was advised for surgery for removal of the lesion. In this context, left parietal craniotomy was performed with total removal of the lesion through intraparietal approach. Reversal from GA and the postoperative period was uneventful and the patient had got rid of clinical symptom completely after surgery.

The tumor was well defined preoperatively and was removed under microscopic surgery meticulously. The patient was very poor to bear the cost of postoperative CT-scan and hence it was not done. The biopsy was sent for histopathological examination. The lesion was nearly spherical measuring 3.0 cm + 2.5 cm, whitish, and firm.

There was no evidence of necrosis. Microscopy revealed multicystic appearance with cyst lined by columnar epithelium with apical mucin. Few goblet cells were seen. Few areas had shown pseudostratified lining epithelium with nuclear hyperchromasia, nuclear enlargement, and increased mitosis.

There was no evidence of stromal invasion. The mucinous material was seen within the lumen. Hemosiderin laden macrophages were present in the stroma. It was confirmed as a lateral ventricular enterogenous cyst. Then the patient was well in around one year. After about one year of the surgical removal of the lesion, there was a recurrence of a headache whose intensity was more than the previous one.

**Figure 2:**
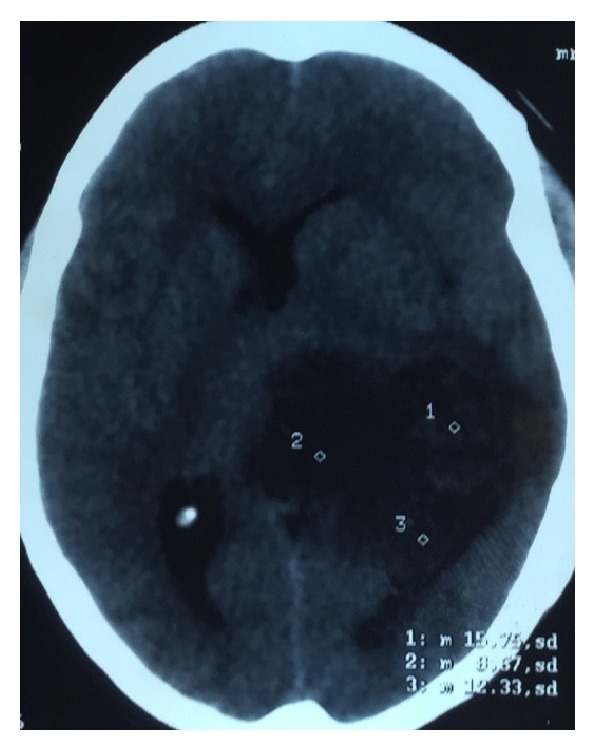
Recurrence of lesion on CT-scan after surgery.

**Figure 3:**
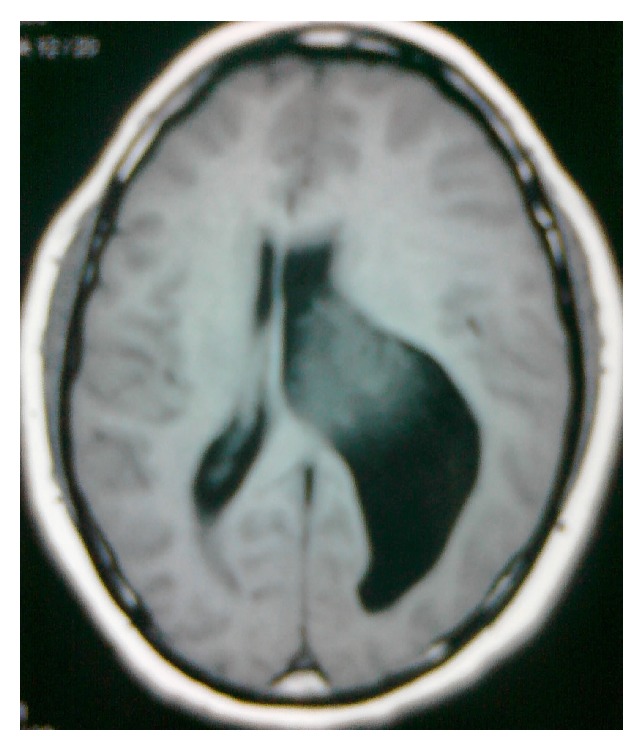
T1 image axial view precontrast film.

**Figure 4:**
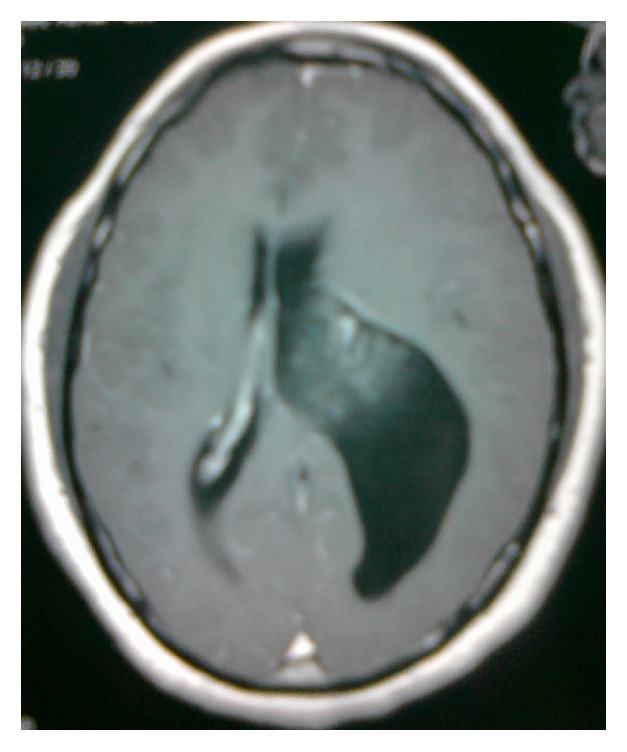
T1 image axial view postcontrast film.

**Figure 5:**
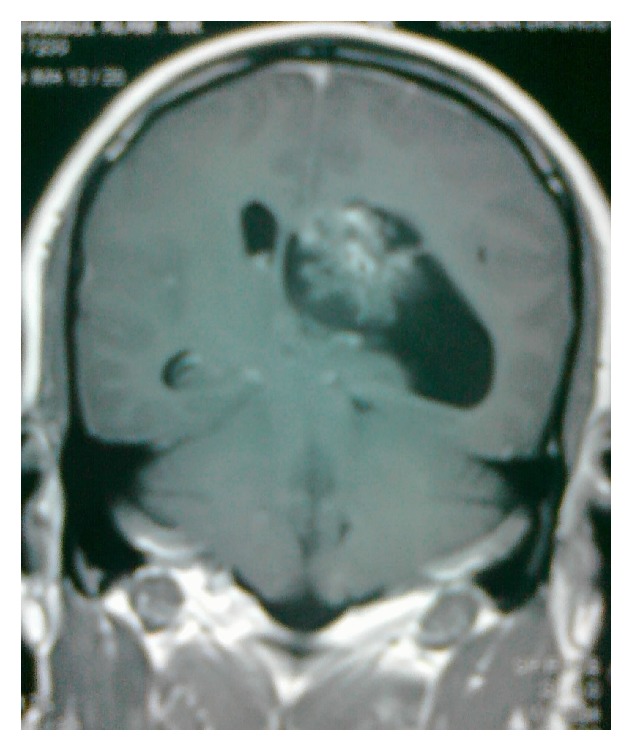
T1 image coronal view postcontrast film.

**Figure 6:**
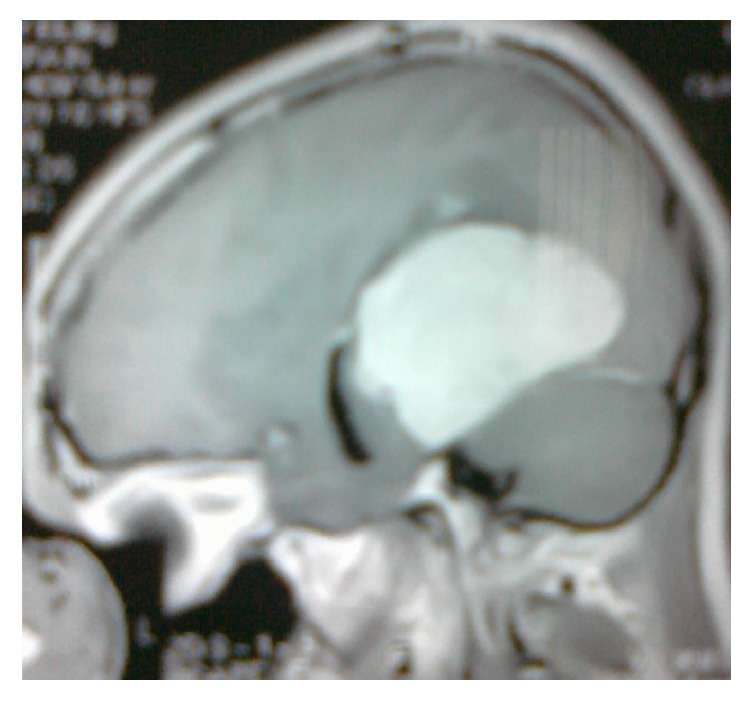
T2 image sagittal view.

**Figure 7:**
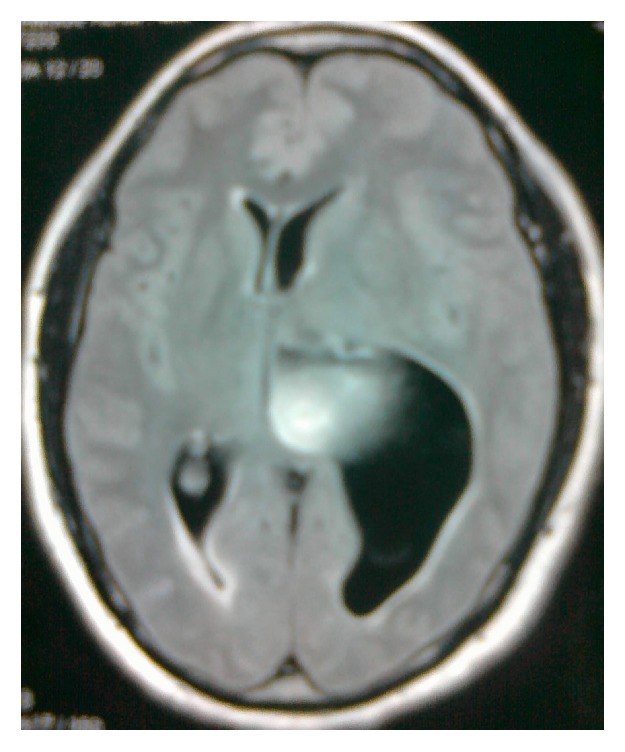
Flare image.

Then the patient was advised to do a CT-scan which revealed lesion of the same pattern in the same location as before (Figure 2). The patient was reexplored surgically and the tumor was removed completely through the same route and histopathology was again revealed it as a case of the enterogenous cyst. Again the total removal of the lesion from the brain was confirmed by postoperative CT-scan. Then the patient was advised for radiotherapy. After completion of radiotherapy, the patient was perfectly well at 13th postoperative month (Figure 2).

## 3. Discussion

CNS cyst is lined by endothelium which is primarily resembling that of GIT. It is also less often similar to respiratory tract which is known as a neurenteric cyst. Neurenteric cysts, colloid cysts, and Rathke cleft are derived from endoderm and these are the endodermal lesions of CNS. Neurenteric cysts have several names which include gastroenterogenous cyst, enterogenous cyst, astrocytoma, enteric cyst, endodermal cyst, and intestinal and archenteric cyst. Most reported enterogenous cysts occur in the spine which is usually ventral to the cord [6, 7]. Association of spinal enterogenous cysts with vertebral abnormalities is also found in 50% of cases [5].

On the other hand, intracranial NECs are usually uncommon with less than 60 cases reported before this series. Most often these intracranial cysts are found in the posterior fossa. These are situated in the midline, anterior to the brainstem, or in the CP angle. These occur in all age groups but supratentorial EC is usually present at a later age, third and fourth decades [1]. With rare exceptions, intracranial NECs have not been associated with bony abnormalities. Supratentorial NECs are rare and so far only 14 cases have been reported [4]. Giant supratentorial neurenteric cysts are rare. Less than 17 cases have been reported in the world literature, the majority of which have been reported in the anterior fossa or in deep midline structures [8].

The precise etiology of the NEC is not known. It has been postulated that these cysts arise at the time of notochordal development at the time of transitory existence of the NE canal. In this situation the notochord as well as the foregut has failed to separate causing primitive endodermal cells to be incorporated into the notochord. This displaced nest of alimentary tissue leads to the cyst. Explanation of the supratentorial cyst cannot be ruled out by this theory; additionally, the posterior glenoid portion of clivus is formed by the cranial aspect of notochord. There are different lesions found in the cranial up to the diverticulum which are behind the oropharyngeal membrane and these may be the embryologic progenitor of midline supratentorial cysts. Again, the above-mentioned theory has failed to explain the midline supratentorial cysts formation [4].

In the spine, it most frequently occurs in spinal canal where these are well recognized but uncommon cause of spinal cord compression. Anatomically these occur most frequently near the junction of cervical and thoracic cord, particularly in young patients [3]. Neurenteric cysts (NECs) are manifestations of a rare congenital dysplasia probably resulting from aspiration of the notochord and the upper gastrointestinal tract. These are most likely of endodermal origin and lined by ciliated and nonciliated low cuboidal with columnar epithelium and contain a few mucin secreting goblet cells. The cysts mostly contain a clear fluid of varying viscosity and protein content. NECs represent 0.01% of CNS tumors [9]. These frequently arise in an intradural extra-axial location anterior to the cervical-thoracic spinal cord while intracranial cysts have a tendency to be infratentorial. Although these are decidedly known to be benign, there have been four reported cases of malignant transformation in cysts which were intracranial which emphasizes the need for gross total resection of the cyst as well as the wall [10, 11].

There is no topographical classification of the enterogenous cyst. These cysts are classified depending on histological characterization of the wall as well as the presence or the absence of components specific to respiratory or gastrointestinal tracts. Simple lining cuboidal or columnar epithelium on a basal membrane with or without cilia is found in type A cysts, resembling gastrointestinal or respiratory epithelium. The presence of other components are usually seen along the gastrointestinal or respiratory tracts which is typical of type B cysts; however, glial or ependymal elements are seen in type C cysts [12].

Nonspecific radiological features make it difficult to differentiate enterogenous cyst from arachnoid cysts, epidermoids, or glioependymal cysts [1]. Enterogenous cysts have high seeding potentials and, therefore, intraoperative confirmation of the histology is very essential to see the complete removal of the cyst. Total excision in a single sitting is the optimum treatment for these cysts; furthermore, it is also mandatory to follow up the patient in a long-term basis which will help to detect any recurrence [12].

## 4. Conclusion

Brilliantly enhancing lateral ventricular mass should be considered endothelial origin. The ideal treatment is complete excision. Proper and adequate management should be taken during surgery, so that spillage of tumor component will be prevented; thus, dissemination and recurrence of the tumor will be occurred.
